# Microbiota-derived tryptophan metabolites in vascular inflammation and cardiovascular disease

**DOI:** 10.1007/s00726-022-03161-5

**Published:** 2022-04-22

**Authors:** Nadja Paeslack, Maximilian Mimmler, Stefanie Becker, Zhenling Gao, My Phung Khuu, Amrit Mann, Frano Malinarich, Tommy Regen, Christoph Reinhardt

**Affiliations:** 1 Center for Thrombosis and Hemostasis (CTH), University Medical Center Mainz, Johannes Gutenberg University Mainz, Langenbeckstrasse 1, 55131 Mainz, Germany; 2 Institute for Molecular Medicine, University Medical Center Mainz, Johannes Gutenberg University Mainz, Langenbeckstrasse 1, 55131 Mainz, Germany; 3 German Center for Cardiovascular Research (DZHK), Partner Site Rhine-Main, Mainz, Germany

**Keywords:** Gut microbiota, Tryptophan metabolism, Endothelium, Vascular inflammation, Hypertension, Atherosclerosis

## Abstract

The essential amino acid tryptophan (Trp) is metabolized by gut commensals, yielding in compounds that affect innate immune cell functions directly, but also acting on the aryl hydrocarbon receptor (AHR), thus regulating the maintenance of group 3 innate lymphoid cells (ILCs), promoting T helper 17 (T_H_17) cell differentiation, and interleukin-22 production. In addition, microbiota-derived Trp metabolites have direct effects on the vascular endothelium, thus influencing the development of vascular inflammatory phenotypes. Indoxyl sulfate was demonstrated to promote vascular inflammation, whereas indole-3-propionic acid and indole-3-aldehyde had protective roles. Furthermore, there is increasing evidence for a contributory role of microbiota-derived indole-derivatives in blood pressure regulation and hypertension. Interestingly, there are indications for a role of the kynurenine pathway in atherosclerotic lesion development. Here, we provide an overview on the emerging role of gut commensals in the modulation of Trp metabolism and its influence in cardiovascular disease development.

## Introduction

The gut microbiota is the assemblage of microbial communities colonizing the intestinal habitat, with the great majority belonging to the bacterial kingdom, but also comprising fungi, archaea, protists, and viruses (Bäckhed et al. 2012). By influencing nutrient availability, through recognition of conserved microbial patterns via a repertoire of innate immune receptors (i.e., Toll-like receptors, nucleotide-binding oligomerization-like receptors, or retinoic acid-inducible gene-I-like receptors), the regulation of a myriad of host metabolic pathways, but foremost all by the uptake of microbiota-derived metabolites, this microbial ecosystem has evolved into a mutualistic relationship with its host, impacting many traits of host physiology (Turnbaugh et al. 2006; Hooper et al. 2012; Koh and Bäckhed 2020; Bäckhed et al. 2005; Schroeder and Bäckhed 2016). For this reason, organisms have to be viewed as holobionts, including their microbiota affecting many organ systems (Meyer-Albich 1950; Margulis and Fester 1991). This is not only exemplified by the evoked adaptive changes in gut morphology or by recent studies on the gut-liver or gut-brain axis (Bayer et al. 2021; de Vadder et al. 2018; Formes et al. 2021; Aswendt et al. 2021), but also by the role of microbiota and its derived metabolites in cardiovascular disease (Karbach et al. 2016).

The pathophysiology of cardiovascular diseases like hypertension and atherosclerosis is closely linked to vascular inflammation (Daiber et al. 2017; Wenzel et al. 2011). Aryl hydrocarbon receptor (AHR)-signaling is well recognized to contribute to those cardiovascular pathologies by inducing expression of pro-inflammatory interleukin (IL)-1β, IL-8, tumor necrosis factor-alpha (TNF-α) and consecutive foam cell formation (Dahlem et al. 2020; Vogel et al. 2004). The various effects of AHR signaling can be conveyed by many different ligands (Safe et al. 2020). Microbiota-derived L-tryptophan (Trp)-metabolites being among them, pathways involved in their generation and signaling deserve attention (Zelante et al. 2013).

**Fig. 1:**
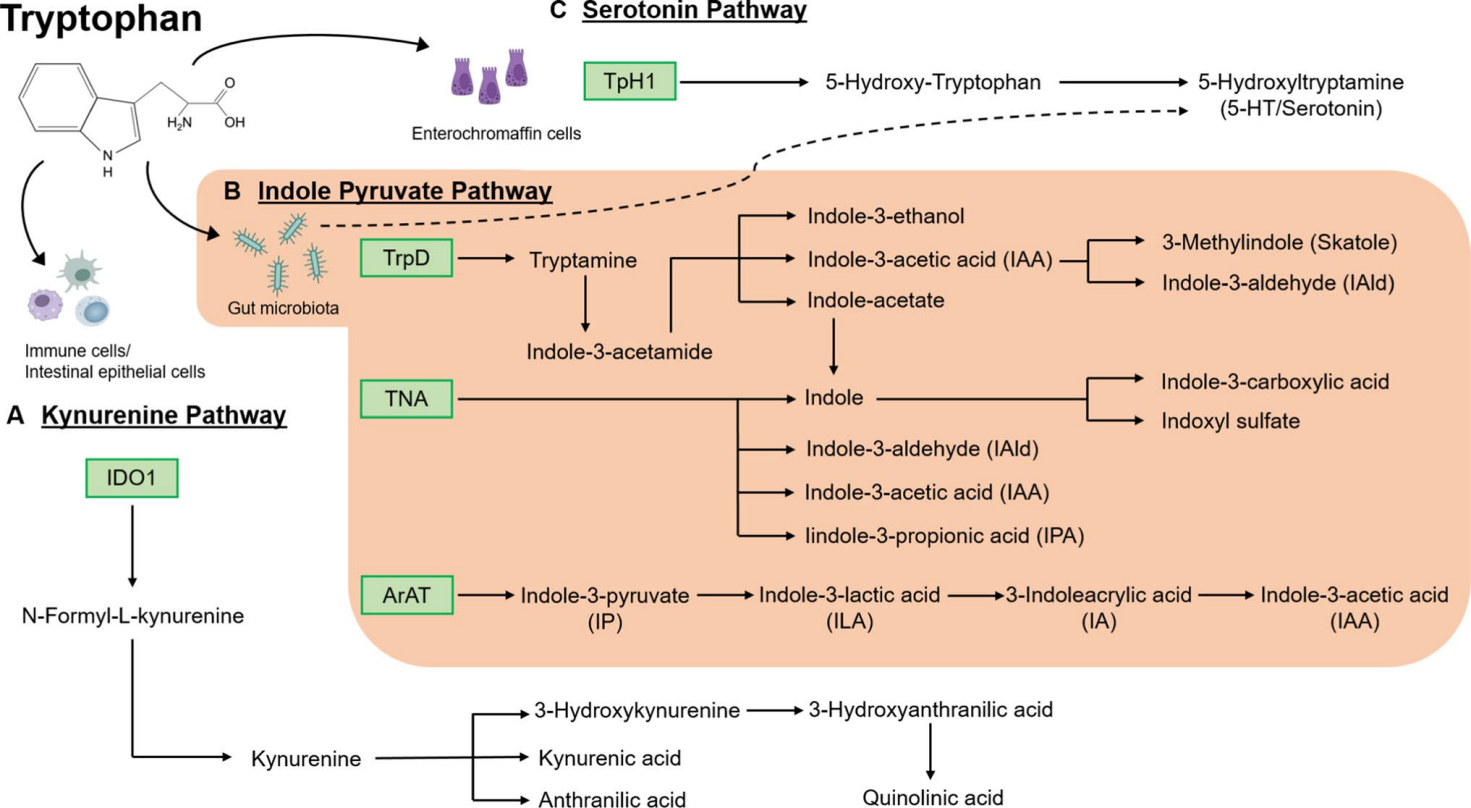
Metabolic pathways of tryptophan. **A** Kynurenine pathway in host immune cells and intestinal epithelial cells. **B** Indole pyruvate pathway performed by gut microbiota. **C** Serotonin pathway performed by enteroendocrine cells. Key enzymes of the pathways are displayed in green: *ArAT* aromatic amino acid transaminase, *IDO1* indolamine-2,3-dioxygenase-1, *TNA* tryptophanase, *TpH1* tryptophan hydroxylase 1, *TrpD* tryptophan dehydrogenase

In the intestine, the bulk of nutritional Trp, an essential amino acid that cannot be synthesized de novo by the mammalian host, is metabolized via the kynurenine pathway in immune cells and intestinal epithelial cells, which is initiated by the enzyme indolamine-2,3-dioxygenase-1 (IDO1), converting Trp to N-formyl-L-kynurenine (Taleb 2019) (Fig. 1). Furthermore, in enterochromaffin cells, the enzyme Trp hydroxylase 1 (TpH1) converts a small part of the nutrition-derived Trp into serotonin (5-hydroxytryptamine, 5-HT). A significant proportion of nutritional Trp enters the indole pathway and is metabolized to tryptamine and signaling-active indole metabolites, involving tryptophanase and decarboxylase enzymes of colonizing gut bacteria (Zelante et al. 2013). Metabolomics analyses demonstrated reduced serum levels of Trp and N-acetyltryptophan in conventionally raised (CONV-R) mice relative to germ-free control mice lacking colonization with a gut microbiota, which is due to bacterial tryptophanase activity, the enzyme catalysing the reaction of Trp to indole, pyruvate, and ammonia (Wikoff et al. 2009). In line, the abundance of tryptamine in the feces of CONV-R mice was shown to be increased by 200% as compared to GF controls (Marcobal et al. 2013). In addition, CONV-R mice displayed elevated serotonin serum levels and the indole-metabolites indoxyl sulfate and indole-3-propionic acid could only be detected in CONV-R mice, indicating that the metabolic capacity of the microbiota acts on nutritional Trp, thus interfering with its availability and functional utilization by the host (Wikoff et al. 2009).

## The gut microbiota as a modifier of tryptophan metabolism—implications for intestinal immune function and pathophysiology

Several bacterial species of the gut microbiota were identified that influence Trp metabolism (Table 1). The synthesis of the monoamine tryptamine is catalysed by tryptophan decarboxylase (TrpD), expressed by gut bacterial species, such as members of the genus Clostridia and Lactobacillus (Agus et al. 2018; Williams et al. 2014). Tryptamine is then further processed by tryptophan-2-monooxygenase (TMO) into indole-3-acetamide, which is converted into indole-3-acetic acid (IAA) and subsequently into indole-3-aldehyde (IAld). In an additional sequence of reactions, the bacterial tryptophanase (TNA), which is expressed by many gram-negative and gram-positive bacteria such as *Escherichia coli*, *Clostridium* sp., and *Bacteroides* sp*.*, converts Trp into indole and its derivatives IAld, IAA, and indole-3-propionic acid (IPA). In the aromatic amino acid aminotransferase and indolelactic acid dehydrogenase–dependent pathway, the enzyme aromatic amino acid transaminase (ArAT) converts Trp into indole-3-pyruvate (IP), which is further metabolized into indole-3-lactic acid (ILA) through the enzyme indolelactic acid dehydrogenase (ILDH). Through the phenyl lactate dehydratase gene cluster (fldAIBC), ILA can further be converted into 3-indoleacrylic acid (IA), which is finally converted into indole-3-propionic acid (IPA) (Williams et al. 2014; Wlodarska et al. 2017). Of note, IPA was revealed to increase blood pressure via cardiac and vascular mechanisms but was negatively associated with advanced atherosclerosis (Konopelski et al. 2021; Cason et al. 2017). Interestingly, it has been demonstrated that this metabolite acts on mitochondrial respiration, with chronic exposure resulting in mitochondrial dysfunction in cardiomyocytes but also in hepatic and endothelial cells (Gesper et al. 2021).

**Table 1:** Microbiota-derived tryptophan metabolites

Tryptophan metabolite	Enzyme	Gut microbes		Reference
		Phylum level	Species level	
Indole	Tryptophanase (TNA)	*Bacteroides* *Clostridium* *Desulfovibrio* *Enterococcus* *Escherichia* *Fusobacterium* *Haemophilus* *Peptostreptococcus*	*Bac. thetaiotaomicron* *Bac. ovatus* *Clo. bifermentans* *Clo. ghoni* *Clo. lentoputrescens* *Clo. limosum* *Clo. malenomenatum* *Clo. sordellii* *Clo. tetani* *Clo. tetanomorphum* *Des. vulgaris* *Ent. faecalis* *Esc. coli* *Fus. nucleatum* *Hae. influenza* *Pep. asscharolyticus*	Devlin et al. (2016) Elsden et al. (1976) Lee and Lee (2010) Smith et al. (1996)
3-Methyl- indole (Skatole)	Indoleacetate decarboxylase (IAD)	*Bacteroides* *Butyrivibrio* *Clostridium* *Eubacterium* *Lactobacillus* *Megamonas* *Parabacteroides*	*Bac. thetaiotaomicron* *But. fibrisolvens* *Clo. bartlettii* *Clo. scatologenes* *Clo. drakei* *Eub. cylindroides* *Eub. rectale* *Lac.* spp. *Meg. hypermegale* *Par. distasonis*	Honeyfield et al. (1990) Russell et al. (2013) Whitehead et al. (2008) Liu et al. (2018)
Indole-3- acetic acid (IAA)	Bacterial Tryptophan Monooxygenase	*Bacteroides* *Bifidobacterium* *Clostridium* *Escherichia* *Eubacterium* *Parabacteroides* *Peptostreptococcus*	*Bac. thetaiotaomicron* *Bac. eggerthii* *Bac. ovatus* *Bac. fragilis* *Bif. adolescentis* *Bif. longum* subsp. *longum* *Bif. pseudolongum* *Clo. bartlettii* *Clo. difficile* *Clo. lituseburense* *Clo. paraputrificum* *Clo. perfringens* *Clo. putrefaciens* *Clo. saccharolyticum* *Clo. sticklandii* *Clo. subterminale* *Esc. coli* *Eub. hallii* *Eub. cylindroides* *Par. distasonis* *Pep. asscharolyticus*	Elsden et al. (1976) Russell et al. (2013) Smith et al. (1996)
3-Indole- acrylic acid (IA)	Phenyllactate dehydratase (fldBC)	*Clostridium* *Peptostreptococcus*	*Clo. sporogenes* *Pep. russellii* *Pep. anaerobius* *Pep. stomatis*	Dodd et al. (2017) Wlodarska et al. (2017)
Indole-3- aldehyde (IAld)	Tryptophanase (TNA)	*Lactobacillus*	*Lac. acidophilus* *Lac. murinus* *Lac. reuteri*	Cervantes-Barragan et al. (2017) Wilck et al. (2017) Zelante et al. (2013)
Indole-3- lactic acid (ILA)	Indole-3-lactic acid dehydrogenase (ILDHase)	*Anaerostipes* *Bacteroides* *Bifidobacterium* *Clostridium* *Escherichia* *Eubacterium* *Faecalibacterium* *Lactobacillus* *Megamonas* *Parabacteroides* *Peptostreptococcus*	*Ana. hadrus* *Ana. caccae* *Bac. thetaiotaomicron* *Bac. eggerthii* *Bac. ovatus* *Bac. fragilis* *Bif. adolescentis* *Bif. bifidum* *Bif. longum* subsp. *infantis* *Bif. longum* subsp. *longum* *Bif. pseudolongum* *Clo. bartlettii* *Clo. perfringens* *Clo. sporogenes* *Clo. saccharolyticum* *Esc. coli* *Eub. rectale* *Eub. cylindroides* *Fae. prausnitzii* *Lac. murinus* *Lac. paracasei* *Lac. reuteri* *Meg. hypermegale* *Par. distasonis* *Pep. asscharolyticus*	Aragozzini et al. (1979) Dodd et al. (2017) Honoré et al. (2016) Russell et al. (2013) Smith et al. (1996) Wilck et al. (2017) Williams et al. (2014) Wlodarska et al. (2017)
Indole-3- propionic acid (IPA)	Acyl-CoA dehydrogenase (ACD) Phenyllactate dehydratase gene cluster (fldAIBC)	*Clostridium* *Peptostreptococcus*	*Clo. botulinum* *Clo. caloritolerans* *Clo. paraputrificum* *Clo. sporogenes* *Clo. cadvareris* *Pep. asscharolyticus* *Pep. russellii* *Pep. anaerobius* *Pep. stomatis*	Dodd et al. (2017) Elsden et al. (1976) Wikoff et al. (2009) Williams et al. (2014) Wlodarska et al. (2017)
Tryptamine	Tryptophan Decarboxylase (TrpD)	*Clostridium* *Ruminococcus*	*Clo. sporogenes* *Rum. gnavus*	Williams et al. (2014)

In recent years, it has become evident that the gut microbiota is an important modifier of host physiology and a driver of various organ pathologies (Schroeder and Bäckhed 2016). Based on germ-free mouse models, recent metabolomics analyses identified a myriad of microbiota-derived metabolites that are readily taken up into the bloodstream and impact on remote organ functions (Wu et al. 2019; Lai et al. 2021). Trp was demonstrated to affect both epithelial immunity as well as gut microbial ecology (Hashimoto et al. 2012). Microbiota-derived Trp metabolites interfere with multiple aspects of host physiology. For instance, microbiota-derived indole modulates the secretion of the incretin hormone glucagon-like peptide-1 (GLP-1) from L-cells of the colonic epithelium (Chimerel et al. 2014).

The indole metabolite indoxyl sulfate is generated in the liver, where it enters the circulation as an albumin-bound serum molecule. Indoxyl sulfate is known to be harmful to various cell types, such as vascular endothelial cells (Hung et al. 2016). Furthermore, it has been reported that dysbiosis of the intestinal microbiota towards a higher abundance of aerobic indole-producing bacteria (e.g. *E. coli*) is associated with the accumulation of the nephrotoxin indoxyl sulfate in the serum of uremic patients, which is due to impaired renal secretion (Deguchi et al. 2002; Takayama et al. 2003). The studies by Takayama et al. showed a significant reduction of indoxyl sulfate serum levels in hemodialysis patients orally treated with non-indole-producing bacteria (e.g. *Bifidobacterium*) by correcting the composition of the gut microbiota (Takayama et al. 2003). In addition, in the gut mucosa, indole metabolites act on the gut epithelial AHR, important to warrant mucosal type 3 innate lymphoid cells (ILC3) and T helper 17 (T_H_17) immunity (Cella et al. 2009; Kiss et al. 2011; Schiering et al. 2017). While this aspect of mucosal immunity is beneficial to protect from infection, the induction of immunity by Trp metabolites comes with the downside of an enhanced susceptibility to autoimmunity (Sonner et al. 2019; Choi et al. 2020).

Inflammatory bowel disease (IBD) is one example of an autoimmune disease that has been linked to changed Trp metabolism (Roager and Licht 2018). It was found that Trp serum levels are significantly decreased in IBD patients, compared to healthy controls, while Crohn’s disease patients have a more severe reduction compared to ulcerative colitis patients (Nikolaus et al. 2017). Also, IAA was shown to be reduced in fecal samples of IBD patients (Lamas et al. 2016). Furthermore, serum concentration of IPA is reduced in patients suffering from active colitis as compared to healthy individuals (Alexeev et al. 2018). Lastly, orally administered indole or IPA was shown to ameliorate colonic inflammation in mice (Alexeev et al. 2018; Whitfield-Cargile et al. 2016). Furthermore, Trp and its metabolites stimulate AHR activity and induce tumor cell proliferation and tumor escape in colorectal cancer (Venkateswaran et al. 2019; Brandacher et al. 2006).

Microbiota-derived metabolites were demonstrated to protect from autoimmune disease development (Rosser et al. 2020). Interestingly, in the mouse experimental autoimmune encephalomyelitis (EAE) model of multiple sclerosis, it was recently demonstrated that the susceptibility to the induction of central nervous system inflammation in IL-17A/F-deficient mice was dependent on the impact that IL-17A exerts on the composition of the gut microbiota (Regen et al. 2021). This highlights the need for a more detailed understanding of how gut commensals interfere with nutritional factors and host metabolism to impact on autoimmune-related disease phenotypes.

In addition to the gut and the nervous system, the vasculature and its endothelial lining are prone to microbiota-dependent inflammatory processes (Karbach et al. 2016; Kiouptsi et al. 2019). Since Trp metabolism is an important determinant of atherogenesis (Metghalchi et al. 2015; Kappel et al. 2020), it is important to understand how the gut microbiota, a known regulator of Trp/indole metabolism, affects vascular inflammatory phenotypes, hypertension, and the development of atherosclerosis.

## Microbiota-derived tryptophan metabolites impacting immune mechanisms

**Fig. 2:**
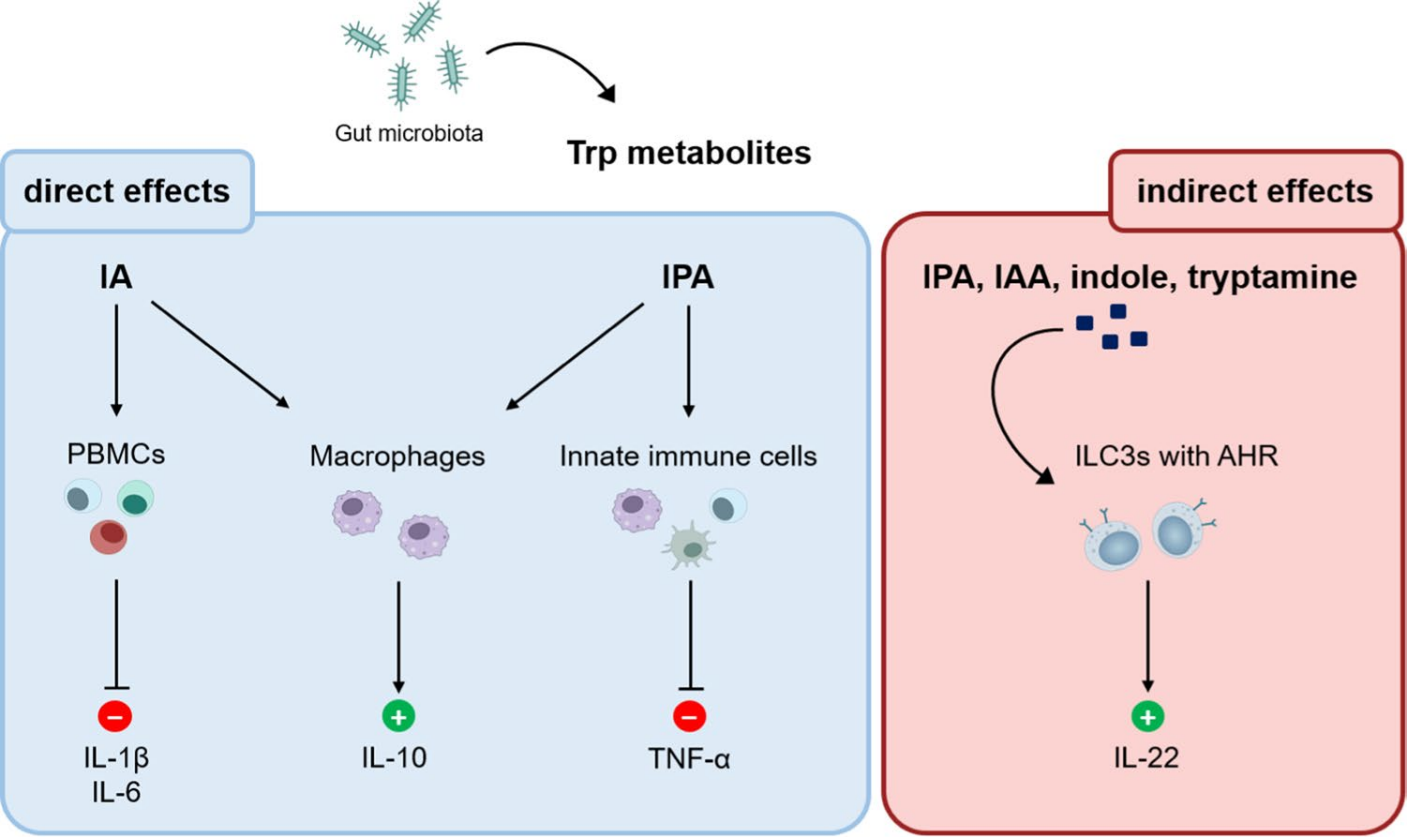
Effects of Trp metabolites on immune cells. Direct (blue box) and indirect effects (red box) of Trp metabolites on immune cells are highlighted. Promoting effects are marked with a green + , repressing effects with a red  – . *AHR* aryl hydrocarbon receptor, *IAA* indole-3-acetic acid, *IA* indoleacrylic acid, *IPA* indolepropionic acid, *ILC3s* innate lymphoid cells group 3, *IL* Interleukin, *PBMCs* peripheral blood mononuclear cells, *Trp* tryptophan, *TNF* tumor necrosis factor

Interestingly, some indole metabolites, such as indole-3-propionic acid (IPA), have direct anti-inflammatory effects on immune cells (Fig. 2) (Wlodarska et al. 2017; Venkatesh et al. 2014). For instance, IPA and 3-Indole-acrylic acid (IA) enhance the production of anti-inflammatory interleukin-10 (IL-10) by macrophages. In addition, IPA reduces the production of pro-inflammatory tumor necrosis factor (TNF) (Wlodarska et al. 2017). Furthermore, IA exerts anti-inflammatory effects by suppressing the expression of the cytokines IL-1β and IL-6 by peripheral blood mononuclear cells (PBMCs) (Fig. 2, left).

However, Trp/indole metabolites also impact immune phenotypes via indirect signaling mechanisms, predominantly mediated through the AHR (Fig. 2, right). The AHR is a transcription factor, which is especially recognized for its role in xenobiotic metabolism (Hubbard et al. 2015a, b). The AHR is expressed on the surface of group 3 innate lymphoid cells (ILC3s) (Cella et al. 2009; Kiss et al. 2011). AHR activation induces IL-22 production (Fig. 2), which also involves TLR2 signaling (Zelante et al. 2013; Crellin et al. 2010). The cytokine IL-22 regulates intestinal mucosal homeostasis and provides resistance to the fungus *Candida albicans* and to rotavirus infection (Zelante et al. 2013; Hernandez et al. 2015). The disturbance of the gut microbiota’s ability to generate AHR ligands was associated with the development of IBD (Lamas et al. 2016). Of note, the AHR can be activated by a wealth of different ligands including Trp/indole metabolites. Upon ligand binding, the AHR undergoes conformational changes to enable its transport from the cytosol into the nucleus (Ikuta et al. 1998). In the nucleus, the aryl hydrocarbon receptor nuclear translocator (ARNT) induces the dissociation of the cytoplasmic complex from the AHR leaving the functional heterodimeric transcription factor complex of AHR/ARNT (Reyes et al. 1992). The AHR/ARNT complex subsequently binds to its target sequence in the DNA and recruits further coactivators to remodel the chromatin and transcribe target genes. The AHR can be activated both, by ligands provided through the kynurenine pathway (therefore by the host), and by microbiota-derived ligands originating from the metabolic Trp pathway. Moreover, the AHR can be activated by dietary constituents or by pollutants. Interestingly, activation of the AHR can either have pro- or anti-inflammatory effects, depending on the ligand or the target cell (Gutierrez-Vazquez and Quintana 2018). Indole metabolites (indole, IPA, IAA, and tryptamine) produced by gut microbiota are known to be AHR activators (Zelante et al. 2014).

The AHR interferes with the immune system by inducing IL-22 production, a cytokine of the IL-10 family that stimulates mucosal defence via the induction of antimicrobial peptides (AMPs) (Taleb 2019; Zelante et al. 2013). IL-22 regulates epithelial cell proliferation and production of antimicrobial peptides (AMPs), decreasing the inflammatory potential of commensal bacteria, which has been shown in metabolic syndrome and atherosclerosis (Fatkhullina et al. 2018; Taleb 2019; Wang et al. 2014). In addition to the regulation of mucosal immune responses, the AHR regulates organogenesis, mucosal barrier function, and cell cycle (Kiss et al. 2011; Hubbard et al. 2015a, b; Gronke et al. 2019). AHR-ligands are cleared and detoxified by cytochrome P450 1 (CYP1) enzymes, having a role in feedback regulation. The in vivo relevance of CYP1 enzymes was demonstrated by the constitutive and restricted expression of Cyp1a1 in intestinal epithelial cells, which resulted in the loss of ILC3s and T helper 17 (T_H_17) cells (Schiering et al. 2017). Hence, gut commensal-derived Trp metabolites interfere with immune regulatory AHR signaling at multiple levels.

## Microbiota-derived tryptophan metabolites in vascular inflammation

Endothelial dysfunction precedes atherosclerosis, one of the most severe forms of vascular inflammation. Analysis of vascular dysfunction in germ-free mouse models indicated that the gut microbiota promotes vascular inflammation and oxidative stress (Karbach et al. 2014). Vascular inflammation is frequently associated with autoimmune diseases such as type 2 diabetes (Steven et al. 2019). Notably, gnotobiotic mouse atherosclerosis models and clinical metagenomic shotgun sequencing studies have firmly established the gut microbiota as a relevant modifier of vascular inflammation and atherosclerosis, the primary cause of myocardial infarction (Karbach et al. 2016; Lindskog et al. 2018; Kiouptsi et al. 2019; Jie et al. 2017; Pontarollo et al. 2020). Interestingly, in a rat model, treatment with different antibiotics resulted in shifts of microbial composition and could ameliorate myocardial infarction, which was paralleled by decreased levels of Trp metabolites like kynurenine, indole acetate, indole propionate, and 3-indoxyl sulfate. At the same time, vancomycin treatment increased serotonin levels (Lam et al. 2016). In recent years, several studies demonstrated that the microbiota’s influence on Trp metabolism can impact on vascular inflammation.

Importantly, the indole pathway promotes the development of vascular inflammatory phenotypes. In vitro experiments with endothelial cells demonstrated that indoxyl sulfate can induce oxidative stress and decrease NO production (Dou et al. 2007; Stinghen et al. 2014; Yu et al. 2011). Furthermore, indoxyl sulfate-treatment led to enhanced pro-coagulant properties of endothelial cells since it increased the expression of the coagulation initiator tissue factor in human umbilical vein endothelial cells (HUVECs) along with elevated tissue factor procoagulant activity on their extracellular vesicles (Gondouin et al. 2013). In addition, indoxyl sulfate was demonstrated to inhibit proliferation and wound healing (Dou et al. 2004). Moreover, in vascular smooth muscle cells, this metabolite was shown to induce proliferation (Yamamoto et al. 2006), tissue factor expression and activity (Chitalia et al. 2013), as well as IL-6 expression (Adelibieke et al. 2014), involving AHR signaling. Importantly, these findings also translated into in vivo studies, where indoxyl sulfate enhanced leukocyte recruitment to the vascular wall and enhanced the release of endothelial microparticles (Ito et al. 2016; Faure et al. 2006). In chronic kidney disease patients, elevated levels of serum indoxyl sulfate correlated with aortic calcification (Barreto et al. 2009). However, recent work suggests that indoxyl sulfate may not be the major contributor to vascular dysfunction associated with ischemic acute kidney injury (Nakagawa et al. 2022).

In contrast to indoxyl sulfate, protective effects were described for indole-3-propionic acid (IPA). IPA could induce pregnane-X-receptor (PXR) expression in the aorta of mice and diminished endothelial-dependent vasodilator responsiveness (Venu et al. 2019). In the context of intestinal inflammation, IPA increased IL-10 receptor (IL-10R) levels in intestinal epithelial cells (Alexeev et al. 2018) and showed an anti-inflammatory effect in murine bone marrow-derived macrophages (Wlodarska et al. 2017). Although IPA administration alone does not seem to be sufficient to protect Western diet-fed mice from detrimental cardiometabolic effects on liver and vasculature (Lee et al. 2020), other in vivo studies suggest that IPA can reduce inflammation (Zhao et al. 2019; Du et al. 2021).

Similar to IPA, the Trp metabolite IAld was also capable of increasing IL-10R expression in vitro and serum levels of both compounds are reduced in mice with active inflammation (Alexeev et al. 2018). In mouse models, IAld treatment resulted in a reduction of the type I interferon response, as shown for graft-versus-host disease and CNS inflammation (Langan, et al. 2021; Swimm et al. 2018; Rothhammer et al. 2016). These observed effects led to the discussion of IAld as a potential treatment option for pulmonary infections with an inflammatory component like aspergillosis (Puccetti et al. 2021). Of note, IAA, another Trp metabolite of the indole pathway produced in bacteria like Clostridium (Elsden et al. 1976), exerted anti-angiogenic effects in HUVECs treated with vascular endothelial growth factor (VEGF) via AHR-signaling (Langan et al. 2021) and was able to inhibit VEGF receptor-2 (VEGFR2) and endothelial nitric oxide synthase (eNOS) phosphorylation in HUVEC cultures (Cerezo et al. 2019). Furthermore, IAA, together with tryptamine, decreased the expression of pro-inflammatory cytokines in cultured macrophages and reduced inflammatory effects of TNF-α in hepatocytes (Krishnan et al. 2018). When mice were fed with a HFD, IDO1 knockout mice had less white adipose tissue, showed lower adiposity, and had lower plasma leptin and LPS levels than WT mice on the same diet (Laurans et al. 2018). Additionally, their livers weighed less, accumulated less lipids and showed lower macrophage infiltration. Overall, this resulted in a lower inflammatory status in the adipose tissue, which was paralleled by higher levels of IAA and lower levels of kynurenine in the intestines of IDO1 knockout mice. Mechanistically, elevations in IAA levels in those mice correlated with elevated IL-22 and IL-17 levels, which led to the hypothesis of IDO1 deletion having a protective function in obesity, insulin sensitivity, and intestinal permeability.

Interestingly, the Indole pathway, through the production of tryptamine by the genera Lactobacillus and Clostridium, was observed to induce serotonin release from enterochromaffin cells in the gut and potentiate inhibitory effects of serotonin on cells in the brain (Takaki et al. 1985; Zucchi et al. 2006). Importantly, the strains *Lactococcus lactis* subsp. *cremoris* (MG 1363), *Lactococcus lactis* subsp. *lactis* (IL1403), *Lactobacillus plantarum* (FI8595), *Streptococcus thermophilus* (NCFB2392), *Escherichia coli* K-12, *Morganella morganii* (*NCIMB, 10,466*), *Klebsiella pneumoniae* (NCIMB, 673) and *Hafnia alvei* (NCIMB, 11,999) were described to produce serotonin directly (O’Mahony et al. 2015). Moreover, the presence of spore-forming gut bacteria was shown to elevate host serotonin levels (Yano et al. 2015). Accordingly, in the study by Wikoff et al*.* it was reported that germ-free mice had a 2.8-fold decrease in plasma serotonin as well as elevated tryptophan levels as compared to conventionalized mice (Wikoff et al. 2009). As serotonin was reported to induce neutrophil degranulation, thus worsening thromboinflammation, the connection between microbiota derived metabolites and serotonin might also be implicated in vascular inflammation (Mauler et al. 2019). Moreover, serotonin is important for central cardiovascular regulation (Ramage and Villalón 2008) and the microbiota was identified to modulate serotonin levels in the brain (Clarke et al. 2013). The ability of Trp metabolites such as IAA and IPA to cross the blood brain barrier might also indicate a central role for those metabolites (Gao et al. 2020).

**Fig. 3:**
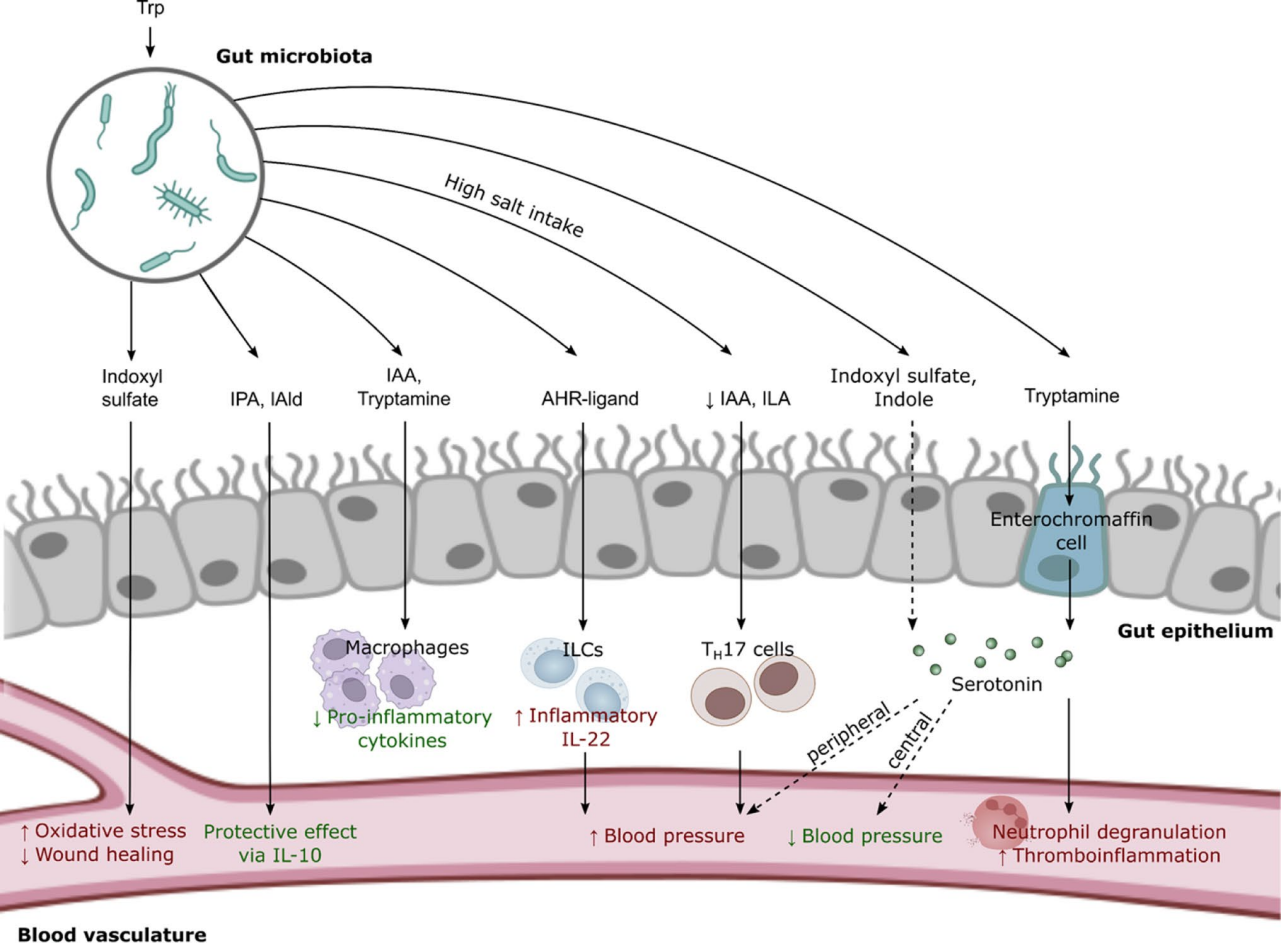
Gut microbiota-derived tryptophan metabolites regulate vascular physiology and disease. Dietary Trp can be converted into various metabolites by the gut microbiota. Indoxyl sulfate induces oxidative stress and inhibits wound healing of the endothelium. Tryptamine initiates the production of serotonin from enterochromaffin cells. Local effects of serotonin include the degranulation of neutrophils, which exacerbates thromboinflammation. Indoxyl sulfate and indole may regulate blood pressure via the serotonin signaling pathway, which acts either through central or peripheral mechanisms. Anti-inflammatory effects are evoked via IPA and lAld following IL‑10 production, or via IAA and tryptamine resulting in a decreased release of pro-inflammatory cytokines from macrophages. Blood pressure is regulated by a variety of AHR ligands that influence intestinal immune cells. Dysbalance of AHR ligands such as IAA or ILA can act on ILCs or T_H_17 cells to alter the blood pressure and promote hypertension. Abbreviations *IAA* indole-3-acetic acid, *AHR* aryl hydrocarbon receptor, *ILA* indole-3-lactic acid, *IPA* indole-3-propionic acid, *IAld* indole-3-aldehyde, *ILC* innate lymphoid cell, *IL* interleukin, *T*_*H*_ T-helper cell, *Trp* tryptophan

The presented literature outlines various roles for Trp metabolites in vascular inflammation (Fig. 3). Indoxyl sulfate was shown to promote a procoagulant state in vitro as well as endothelial dysfunction in vivo. IPA, IAld and IAA on the other hand are predominantly reported to exert anti-inflammatory responses. Serotonin release also seems to be modulated by the microbiota-derived Trp metabolite Tryptamine, which might affect cardiovascular disease development. Yet, conclusive mechanisms by which these metabolites operate remain to be elucidated and would be highly beneficial for their further characterization. This is also highlighted by conflicting reports on the role of IDO1 in these pathologies, as not only direct effects of its substrates have to be considered, but also their absence and shifts in other regulatory pathways. Nevertheless, Trp metabolites impact on vascular inflammation, which has been firmly linked to hypertension (Wenzel et al. 2011).

## Microbiota-derived tryptophan metabolites in hypertension

Hypertension is a common and serious medical condition that can lead to cardiovascular disease. Elevated blood pressure significantly increases the risks of several organ pathologies in heart, brain, or kidney, leading to major complications such as congestive heart failure, cerebral haemorrhage, and renal failure (Doyle 1991; Price and Kasner 2014). Approximately 1.28 billion adults worldwide are affected by hypertension, with an estimated 46% of all affected individuals being unaware of their condition (World Health Organization 2021; https://www.who.int/news-room/fact-sheets/detail/hypertension).

High blood pressure can be caused by several factors: The non-influenceable risk factors, such as genetic predisposition, age or pre-existing diseases like diabetes or kidney diseases, and the influenceable risk factors, which can be affected by lifestyle and environment (Oliveras and La Sierra 2014; Ondimu et al. 2019). In particular physical inactivity, high alcohol and tobacco consumption, stress, obesity, and unhealthy diets, such as the Western diet (including high salt and sugar intake, hyper‑consumption of saturated fats and trans-fats and low levels of nutrition-dense foods like fruits and vegetables), increase the risk of high blood pressure (Narkiewicz 2006; Ondimu et al. 2019; Ruivo and Alcântara 2012; Ozemek et al. 2018). Hypertension is thus related to food intake, which in turn is closely linked to microbiota (Derer et al. 2017; Rothschild et al. 2018). For example, high salt intake affects the gut microbiome by depleting particular bacterial species such as *Lactobacillus murinus*. The presence of Lactobacillus has been shown to prevent the formation of T_H_17 cells and consequently reduce hypertension in mice. Lactobacillus depletion due to increased salt intake was accompanied by decrease in the Trp metabolites IAA and ILA, suggesting a link between Trp metabolites and cardiovascular health (Wilck et al. 2017). Furthermore, gut microbiota drives hypertension via the blood pressure hormone angiotensin‑II by promoting vascular inflammatory processes mediated by monocyte chemoattractant protein‑1 (MCP-1, CCL2) and IL‑17 (Karbach et al. 2016). In this study, germ-free housing conditions resulted in the protection against angiotensin II-induced hypertension and associated organ damage.

Since diet is known to influence the composition of the gut microbiome (Derer et al. 2017), it is not surprising that recent mechanistic studies linked the microbiota to hypertension. Most interestingly, hypertension was found to be transferable by fecal microbiota transplant (FMT) into the germ-free mouse model (Li et al. 2017), demonstrating a causal role of the microbiota. The presence or absence of different gut microbes was associated with high blood pressure. For instance, the absence of gram-negative bacteria such as Klebsiella, Parabacteroides, Desulfovibrio, and Prevotella has been linked to elevated blood pressure (Verhaar et al. 2020). Moreover, people affected by hypertension were reported to have a reduced abundance of the genus Oscillibacter and Lactobacillus (Dan et al. 2019), microbes associated with Trp metabolism (Roager and Licht 2018; Chen et al. 2019). Therefore, microbiota-derived Trp metabolites seem to play a role in blood pressure regulation.

The effects of cardiac pressure overload on gut dysbiosis were studied in a transverse aortic constriction (TAC)-model. Within this study, elevated blood pressure was found to decrease Lactobacillus species involved in gut homeostasis and Trp metabolism (Carrillo-Salinas et al. 2020; Larigot et al. 2018). Moreover, during the TAC-induced cardiac pressure overload a reduction in the cardiac expression of the AHR, a known receptor for Trp ligands, was found in CONV-R mice but not in mice lacking the microbiome (Carrillo-Salinas et al. 2020). This supports the conclusion that hypertension is influenced by microbiota-derived Trp metabolites. Besides that, diet-derived AHR ligands promote the production of the inflammatory mediator IL-22 in the gastrointestinal tract (Lee et al. 2012), which in turn leads to increased blood pressure and endothelial dysfunction (Ye et al. 2017). This indicates the importance of the local activity of certain Trp metabolites.

In the small intestine, the essential amino acid Trp is converted into indole and its related derivatives such as IAA, IPA and ILA by various intestinal bacteria (listed in Table 1) (DeMoss and Moser 1969; Donia and Fischbach 2015; Konopelski and Ufnal 2018). Several studies indicate an effect of indole and other Trp-derived indole-compounds on gastrointestinal and circulatory system function, including conditions such as hypertension (Hubbard et al. 2015a, b; Huć et al. 2018). In rats, colonic indole has been reported to increase portal blood pressure, thereby affecting processes of intestinal inflammation and hemostasis, most likely via influencing the function of the gut-vascular barrier (Huć et al. 2018; Jaworska et al. 2017). The gut-vascular barrier was described as a permeable barrier for small molecules whose permeability can be influenced by microbiota such as *Salmonella typhimurium* (Spadoni et al. 2015). According to this, portal hypertension increases the permeability of the microbiota-derived indoles from the intestine into the blood circulation (Huć et al. 2018).

Targeting the gut-vascular barrier is only one possible way how Trp metabolites can influence the circulatory system. The regulation of blood pressure underlies a variety of mechanisms mediated peripheral and central by the brain (Dampney et al. 2002; Guyton et al. 1972), in which microbiota-derived metabolites may also play a role (Tomasova et al. 2016; Verhaar et al. 2020). For instance, the Trp metabolites indole and indole sulfate are able to influence blood pressure via both pathways. Huć et al. discovered the effect of the two metabolites on the circulatory system in rats (Huć et al. 2018). Therefore, indole and indole sulfate were administered intravenously and into the cerebroventricular system at different concentrations to investigate their central and peripheral effects on heart rate and blood pressure. Upon intravenous administration, both indole and indoxyl sulfate increased mean arterial blood pressure at different concentrations. However, significant changes in heart rate occurred with indoxyl sulfate treatment. During cerebroventricular administration of the two metabolites, a significant decrease in mean arterial blood pressure as well as a decrease in heart rate were observed with indole treatment but not with indoxyl sulfate. This highlights the regulatory role of the microbiota-derived Trp metabolites on peripheral and central blood pressure mechanisms (Huć et al. 2018; Gao et al. 2020). In particular, indole and indoxyl sulfate affect arterial blood pressure via the serotonin signaling pathway since pre-treatment with serotonin-receptor blockers such as ondansetron and pizotifen inhibited the hemodynamic effects of indole and indoxyl sulfate (Huć et al. 2018).

Overall, there is multiple evidence linking Trp and its microbiota-derived metabolites with blood pressure regulation and hypertension (Fig. 3). Besides central and peripheral regulation by microbiota-derived Trp metabolites via the serotonin pathway, local activation of AHR signalling by indole metabolites in the gut, but also impaired gut-vascular barrier function could be involved.

## Microbiota-derived tryptophan metabolites in atherosclerosis

Despite the use of cholesterol-lowering therapies to reduce atherosclerosis, nearly 18 million people die each year from CVD (Roth et al. 2017). Atherosclerosis is a chronic inflammatory disease, affecting both large and medium sized arteries, initiated by vascular inflammation, increased endothelial cell permeability, and intimal low-density lipoprotein (LDL) cholesterol accumulation (Theodorou and Boon 2018). Overgrowth of atherosclerotic plaques in coronary arteries can result in myocardial ischemia, which leads to myocardial cell death and acute myocardial infarction (AMI) (Thygesen et al. 2018; Ibanez et al. 2018).

Inflammation is considered a key driver of arterial thrombotic events (Ross 1999; Hansson 2005; Libby et al. 2009). Several amino acid metabolic pathways were identified as checkpoints for the control of inflammation-related mechanisms. For example, the branched-chain amino acids (leucine, isoleucine, and valine) have been shown to promote endothelial cell dysfunction by increasing production of reactive oxygen species (ROS) and inflammation (Zhenyukh et al. 2018). In particular, Trp metabolites were implicated in cardiovascular disease (Nitz et al. 2019; Kappel et al. 2020). For example, in clinical studies on advanced atherosclerosis, Trp was negatively associated and the kynurenine/Trp ratio was positively associated with advanced atherosclerosis (Pedersen et al. 2015). In the apolipoprotein E (*Apoe)*-deficient mouse atherosclerosis model, antibiotic treatment resulted in increased atherosclerosis, connected to a loss of intestinal microbiome diversity and alterations in microbial metabolic functional capacity with a major impact on the host serum metabolome (Kappel et al. 2020). Pathways that were modulated by antibiotics and connected to atherosclerosis included diminished tryptophan and disturbed lipid metabolism. In support of the role of reduced microbial tryptophan biosynthesis in antibiotics-induced atherosclerosis, supplementation of tryptophan in the diet was able to partially reduce atherosclerotic lesion size in antibiotics-treated *Apoe*-deficient mice.

**Fig. 4:**
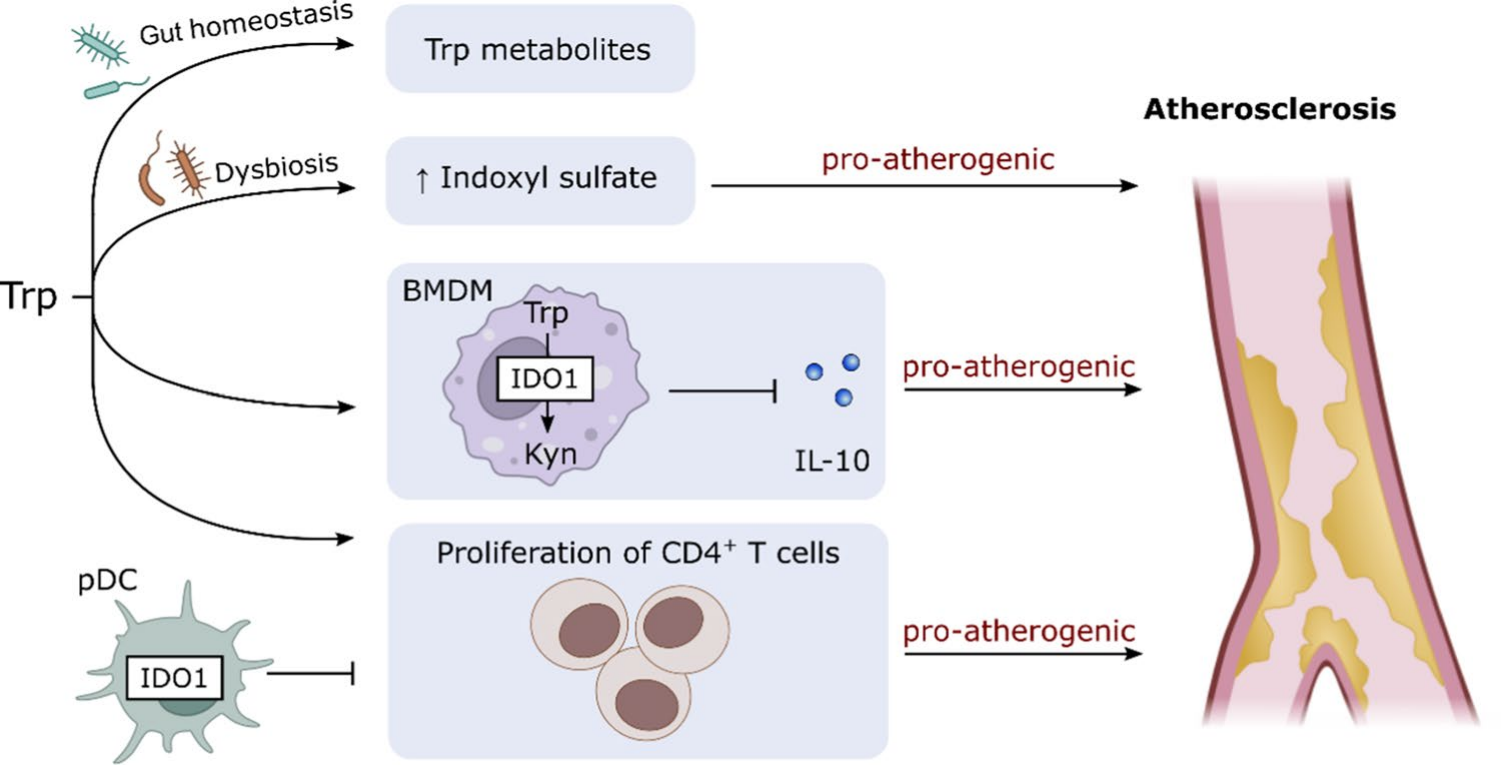
Dysbalance in tryptophan (Trp) metabolism promotes atherosclerosis. Gut dysbiosis increases the production of indoxyl sulfate which may contribute to atherogenesis. Absence of microbiota promotes Trp involvement in the kynurenine pathway. BMDMs process Trp via IDO1 into Kyn metabolites. The decreased release of IL-10 has a pro-atherogenic effect. Accumulation of CD4^+^ T cells is crucial for the progression of atherosclerosis and is promoted by Trp. pDCs are known for their protective role in atherosclerosis by inhibiting CD4^+^ T cell proliferation. Abbreviations: *BMDM* bone marrow-derived macrophage, *CD* cluster of differentiation, *IDO1* indolamine-2,3-dioxygenase-1, *IL* interleukin, *Kyn* kynurenine, *pDC* plasmacytoid dendritic cell, *Trp* tryptophan

IDO is a rate limiting enzyme implicated in Trp catabolism via the kynurenine pathway (Higuchi and Hayaishi 1967; Yamamoto, and Hayaishi 1967). During inflammation, IDO is up-regulated mostly in macrophages and dendritic cells by pro-inflammatory stimuli, such as IFN-γ (Chon et al. 1996). Daissormont et al. reported a protective effect of plasmacytoid dendritic cells (pDCs) in a model of atherosclerosis, proposing that pDC depletion was accompanied by increased CD4^+^ T cell proliferation, interferon-γ expression by splenic T cells, and plasma interferon-γ levels. Lymphoid tissue plasmacytoid dendritic cells from atherosclerotic mice showed increased IDO expression and IDO blockage abrogated the pDC suppressive effect on T-cell proliferation (Daissormont et al. 2011). In vascular inflammation and atherosclerosis, IDO1 activity in bone marrow-derived macrophages was proposed to have adverse effects by inhibition of the anti-inflammatory cytokine IL-10 via kynurenic acid mediated activation of a cAMP-dependent pathway and inhibition of Erk1/2 phosphorylation (Metghalchi et al. 2015). As macrophages are crucially involved in vascular inflammation and the pathogenesis of atherosclerosis (Shirai et al. 2015), changes in IDO1 activity might play a role in these diseases as well. A recent study on *Apoe*-deficient mice suggested that IDO1 protects against the development of atherosclerotic lesions as *Indo*-knockout mice deficient in *Apoe* had an increased lesion area, increased macrophage and CD4^+^ T cell content in their atherosclerotic lesions, and reduced IL-10 production in B cells (Fig. 4) (Cole et al. 2015). Moreover, by depriving T cells of Trp, IDO activity was hypothesized to regulate T cell-related immunity (Fallarino et al. 2006) and therefore to decrease vascular inflammation and the progression of atherosclerosis (Niinisalo et al. 2010). Indeed, recent work indicates that IDO is critically involved in the regulation of intestinal Trp metabolism, thus impacting on the microbiota-dependent control of metabolic disease (Laurans et al. 2018).

In patients with coronary artery disease, IDO activity was associated with worse cardiovascular outcome (Pedersen et al. 2011, 2015; Eussen et al. 2015). Furthermore, in patients with end-stage renal disease, IDO activity was linked to atherosclerosis progression (Pawlak et al. 2009). In line, kynurenine supplementation was associated with an aggravation of cardiac function as well as a deleterious cardiac remodeling as revealed by lower capillary number, increased infarct size and interstitial fibrosis. IDO inhibitor treatment, as well as total and specific endothelial deletion of IDO, was recently shown to protect against the deleterious cardiac effects of MI-induced ischemia (Melhem et al. 2021). In contrast, kynurenine supplementation, a major IDO-related metabolite, abolished the protective effects of IDO deficiency in this setting. Hence, the inhibition of IDO activity might represent a novel potential therapeutic strategy to mitigate ischemic cardiac injury.

Of note, dysbiosis of the gut microbiota has been shown to have an adverse role in the development of atherosclerosis by increasing the production of indoxyl sulfate from indole and promoting the progression of chronic kidney disease (CKD) (Poesen et al. 2016). Due to the reduced clearance capability of the kidneys, indoxyl sulfate is accumulated and contributes to characteristic phenotypes of atherosclerosis such as endothelial dysfunction (Dou et al. 2004) and coronary calcification (Adijiang et al. 2008).

Taken together, these recent results from clinical and experimental studies highlight the importance of mechanistic investigations on the microbe-host interactions interfering with Trp metabolism, which may contribute to atherosclerosis and its end-stage complications.

## Concluding remarks

The metabolic capacity of the gut microbiota is increasingly recognized to impact on cardiometabolic and cardiovascular disease phenotypes (Kiouptsi et al. 2020; Vieira-Silva et al. 2020). Dependent on the nutritional availability of the essential amino acid Trp, bacteria of the gut microbiota influence host serotonin biosynthesis in enterochromaffin cells and certain members of this microbial ecosystem are even capable to directly provide serotonin to their host (Yano et al. 2015; O’Mahony et al. 2015). Indeed, the microbiota-induced elevation of serotonin levels by spore forming bacteria might contribute to vascular inflammation, most likely by its impact on neutrophil function (Mauler et al. 2022). Another Trp-dependent pathway modulated by the gut microbiota is the indole pathway, which acts via the AHR, thereby impacting mucosal ILC3 and T_H_17 immunity (Cella et al. 2009; Kiss et al. 2011; Schiering et al. 2017). Although the Trp catabolic kynurenine pathway is metabolically independent of the enzymatic repertoire of the gut microbiota, still this pathway underlies the regulation by microbiota-regulated type I interferons (Chon et al. 1996; Schaupp et al. 2020). Of note, there is ample evidence for a protective role of the gut microbiota in atherosclerotic lesion development, which is for instance mediated by its cholesterol-lowering effects (Pontarollo et al. 2020). Importantly, in mouse atherosclerosis models, a protective role of dietary tryptophan has been demonstrated (Kappel et al. 2020).

In conclusion, there is comprehensive experimental and clinical evidence for the involvement of microbiota-related Trp metabolites in vascular inflammation, blood pressure regulation and cardiovascular disease development. Hence, future work should focus on the pro- and anti-inflammatory effects of microbiota-derived Trp metabolites and their direct and indirect impact on host immune cells. Another future challenge is to dissect effects arising from microbiota-produced Trp metabolites from the various influences of endogenously produced metabolites of the kynurenine pathway. Furthermore, using well-defined cell-type specific mouse models, it will be interesting to define how these pathways interfere to regulate host (patho)physiology on the molecular and cellular level.

## Declarations

### Research involving human and animals participants

No research involving humans or animals was carried out.
